# Natural agents mediated autophagic signal networks in cancer

**DOI:** 10.1186/s12935-017-0486-7

**Published:** 2017-11-28

**Authors:** Eun Jung Sohn, Hwan Tae Park

**Affiliations:** 1 College of Korean Medicine, Kyung Hee University, 1 Hoegi-dong, Dongdaemun-gu, Seoul, 130-701 Republic of Korea; 2 Peripheral Neuropathy Research Center, Department of Physiology, College of Medicine, Dong-A University, Dongdaesin-Dong, Seo-Gu, Busan, 602-714 Republic of Korea

**Keywords:** Autophagy, MicroRNA, Natural agents

## Abstract

Recent studies suggested that natural compounds are important in finding targets for cancer treatments. Autophagy (“self-eating”) plays important roles in multiple diseases and acts as a tumor suppressor in cancer. Here, we examined the molecular mechanism by which natural agents regulate autophagic signals. Understanding the relationship between natural agents and cellular autophagy may provide more information for cancer diagnosis and chemoprevention.

## Background

Autophagy, also called type II programmed cell death, is a catabolic process that leads to degradation of targeted organelles or cellular proteins. The first stage of the autophagic process is the formation of autophagosomes arising from intracellular membrane structures, such as the endoplasmic reticulum (ER), plasma membrane [1, 2], Golgi or endosome [1], and the ER‒mitochondrial contact site called the mitochondrion-associated ER membrane [2, 3].

The serine/threonine protein kinase target of rapamycin (TOR) acts as a central inhibitor of autophagy. In yeast, TOR inhibition mediates the kinase activity of autophagy-related gene (Atg) 1 and enhances the Atg1 binding affinity to Atg13 and Atg17 to initiate autophagosome formation [4]. The mammalian Atg13/focal adhesion kinase (FAK) family interacting protein of 200 kD (FIP200)/UNC-51-like kinase complex mediates mechanistic target of rapamycin (mTOR)-mediated autophagy induction [5, 6]. Multiple Atg proteins are required to form the phagophore in autophagosome formation. To initiate the phagophore, the membrane recruits PtdIns3K vacuolar protein sorting 34, the myristoylated serine/threonine kinase Vps15 (p150), Beclin 1, and Atg14 [6–9]. Next, the elongation and expansion stages require a ubiquitin-like system such as the ATG5/ATG12/ATG16L multimeric complex and microtubule-associated protein light chain 3 (LC3) processing [10]. Finally, when the autophagosome is mature, it fuses with the lysosome to form the ‘autolysosome’ to degrade organelles or cellular proteins.

Autophagy plays a role in several biological processes, such as aging, development, and differentiation [11, 12], and it is regulated by 5′ AMP-activated protein kinase (AMPK) and mTOR downstream of PI3 kinase/AKT, which control cell growth and protein synthesis [13]. When growth factors are withdrawn, the autophagosome forms to induce autophagy [14].

## Autophagy and cancer: anti-tumorigenic effects of autophagy

Autophagy is both inhibitory and beneficial to malignant transformation [15, 16]. Mukubou et al. showed that enhanced sensitivity of pancreatic cancer cells to gemcitabine or ionizing radiation treatment activated autophagic flux [17]. Interference in the autophagic machinery in heterozygous or homozygous knockout mouse models resulted in oncogenesis [18]. Beclin 1, which regulates the initiation stages of autophagosome formation, plays a role as a tumor suppressor. Beclin 1^−/−^ mice displayed tumorigenesis and altered early embryonic development [19]. Beclin 1^+/−^ immortalized baby mouse kidney (iBMK) epithelial cells promoted tumorigenesis.

There is evidence that disruption of Atgs promotes tumor cell death induced by anti-cancer drugs, such as histone deacetylase inhibitors, temozolomide, and etoposide [20, 21]. Guo et al. showed that Atg7-deficient lung tumors with *p53* deletion resulted in lipid accumulation [22]. Pyo et al. [23] reported that Atg5 regulates interferon-γ-induced autophagic cell death by interacting with Fas-associated protein with death domain, which is a key signaling molecule involved in cell death.

## Autophagy and cancer: pro-tumorigenic effects of autophagy

Autophagy is constitutively activated in cancer cells, especially RAS-driven tumor cells [24, 25], and transcriptional control of autophagy- and lysosome-related genes induces pancreatic cancer metabolism [18]. Wei et al. [26] demonstrated that deletion of FIP200, which plays a role in autophagy, inhibits mammary tumorigenesis by impairing tumor cell proliferation in vivo in an oncogene-driven tumor model. There are several reports that accumulation of p62, which is a marker of autophagic flux, affects tumorigenesis and/or tumor progression. For instance, expression of p62 is highly induced in non-small-cell lung cancer [27] and breast cancer [28], and p62/SQSTM1-knockout mice showed inhibition of RAS-induced tumorigenesis compared with wild-type animals [29].

## Autophagy and apoptosis

**Fig. 1:**
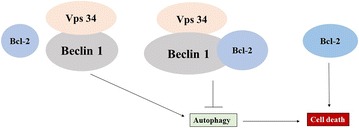
The scheme of Beclin 1 and Bcl2 interaction. Beclin 1 forms a protein complex with VPS34. When Beclin 1 interacts with Bcl-2, Beclin1–Bcl-2 complex inhibits autophagy. However, when Beclin1–Bcl-2 complex dissociate, autophagy initiates

Beclin 1/Atg six is an essential component involved in formation of autophagic vesicles [30]. As shown in Fig. 1, Beclin 1 interacts with Bcl-2, which is an anti-apoptotic protein [31, 32], and inhibits autophagy. The interaction between Beclin 1 and Bcl-2 is via a BH3 domain in Beclin 1 [32, 33], and interference of this interaction was found to enhance autophagy [32].

There is evidence that caspases play important roles in autophagy and apoptosis [34]. Oral et al. [35] reported that caspase-8 overexpression resulted in Atg3 cleavage and thereby its degradation. Caspase 9 interacts with Atg7, and Caspase9–Atg7 complexes enhanced LC3II activity [35]. Zhu et al. [36] reported that caspase-3 plays an important role in autophagy via cleavage of Beclin-1 at positions 124 and 149. Therefore, crosstalk between autophagy and apoptosis may provide important information for cancer therapy.

## Natural agents as modulators of autophagic signals

**Fig. 2:**
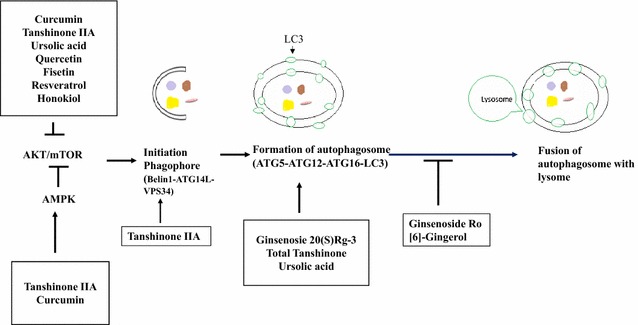
The autophagic signal by natural agents. Curcumin, Tanshinone IIA, ursolic acid, quercetin, fisetin, resveratrol, and honokiol inhibit AKT/mTOR pathway. Tanshinone IIA and curcumin also enhance activation of AMPK which inhibits AKT/mTOR pathway. Tanshinone IIA modulates the initiation of phagophore formation. Ginsenoside 20(S) Rg-3, total tanshinone and ursolic acid enhance the formation of autophagosomes. Ginsenoside RO and [6]-Gingerol inhibit autophagosome–lysosome fusion

Several studies have revealed the therapeutic effectiveness of drug re-positioning of existing drugs and the use of newly developed drugs for the treatment of disease and tumors [37–39]. Natural agents have emerged as novel therapeutic agents of drug-repositioning to influence autophagic activity [3]. As shown in Fig. 2, several natural agents modulate autophagy. For example, tanshinone IIA [40], ursolic acid [41], quercerin [42], fisetin [43], resveratrol [44], and honokiol [45] act as inhibitors of the AKT/mTOR pathway. Tanshinone IIA modulates the initiation of phagophore formation. Ginsensoside [46] and ursolic acid [47] affect the formation of autophagosomes. Ginsenoside RO inhibits autophagosome–lysosome fusion [48].

## Resveratrol

Resveratrol, a polyphenol phytoalexin found in grapes, possesses anti-cancer [49, 50] and antioxidant functions [51]. Resveratrol induced apoptosis and autophagy in ovarian cancer cells via inactivation of signal transducer and activator of transcription signaling [52] and induced autophagic cell death in prostate cancer cells via regulation of stromal interaction molecule 1 [53]. It also induced autophagy via downregulation of the Wnt/β-catenin signaling pathway in breast cancer stem-like cells [54]. Ge et al. [44] reported that resveratrol induced autophagy and apoptosis via repression of AKT/mTOR/p70S6K/4E-BP1 and enhancement of p38 MAPK signaling in T cell acute lymphoblastic leukemia cells.

## Apigenin

Apigenin (4′,5,7-trihydroxyflavone), a naturally occurring flavone found in many fruits (apples, grapes), vegetables (onions, parsley), and tea, was shown to have chemopreventive effects in vitro and in vivo [55, 56]. Lee et al. [57] showed that apigenin induced autophagy in HCT116 human colon cancer cells, and when combined with 3MA, which inhibits autophagy, it increased apigenin-induced apoptosis in HCT116 cells. Autophagy inhibition enhanced apigenin-induced apoptosis in breast cancer T47D cells [58]. Apigenin induced autophagic cell death via reactive oxygen species (ROS) production and accumulation of G2/M cell cycle arrest in human papillary thyroid carcinoma cells [59].

## Ursolic acid

Ursolic acid, a pentacyclic triterpenoid known for its anti-tumor effects [60, 61], increased the expression of LC3-II, an autophagosome marker, and induced autophagy via the Beclin-1 and AKT/mTOR pathways. In addition, treatment with 3-methyladeninet or Beclin-1/Atg5 siRNAs enhanced ursolic acid-mediated cell cytotoxicity in prostate cancer cells [62]. Xavier et al. [47] reported that ursolic acid induced accumulation of both LC3-II and p62 by activation of JNK in HCT15 cells. Zhao et al. [63] demonstrated that ursolic acid treatment induced autophagy via eukaryotic translation initiation factor 2-α kinase 3 activity, implying that ER stress is an important factor in autophagy. Leng et al. also demonstrated that ursolic acid treatment led to apoptosis in cervical cancer TC-1 cells and, according to transmission electron microscopy, resulted in autophagic vacuoles. siRNA-mediated knockdown of Atg5 combined with ursolic acid treatment in TC-1 tumor cells attenuated LC3 II accumulation, as shown by Western blotting, implying that the ursolic acid-induced autophagy relies on Atg5 [64].

## Tanshinone

Tanshinones (tanshinone I and IIA) are derived from a traditional Chinese herb and are well-known bioactive herbal compounds with chemopreventive properties [65]. Tanshinone IIA activated LC3 II expression in various cancer cells, such as prostate and gastric cells [66, 67]. It also induced apoptosis and autophagy via the mTOR/p70S6K/AMPK pathway in KBM-5 leukemia cells [68]. Gao et al. [40] reported that treatment of 95D cells with total tanshinones upregulated the expression of autophagic proteins, such as Atg3, Atg5, Atg7, Atg12, Beclin-1, and LC3II, in a dose-dependent manner. Jing et al. demonstrated that tanshinone treatment of gastric cancer cells, such as BGC823 and SGC7901 cells, induced autophagy and attenuated tumor proliferation in nude mice bearing BGC823 tumor xenografts [66]. Tanshinone II treatment in human osteosarcoma MG63 cells upregulated Beclin, which is involved in the formation of autophagic vesicles, inducing autophagy and apoptosis via ROS [69].

## Curcumin

Curcumin, a hydrophobic polyphenol, possesses anti-cancer functions in various human cancer cells, such as pancreatic and prostate cancer cells [70, 71]. Zhang et al. [72] reported that curcumin treatment of colon cancer HCT116 cells induces autophagy via inhibition of the AKT/mTOR signaling pathway and upregulation of the transcriptional activity of the transcription factor EB. Guan et al. [73] also demonstrated that curcumin treatment induced autophagy via activation of AMPK and suppressed the proliferation and migration of MDA-MB-231 breast cancer cells. Curcumin induced autophagy by upregulating the AMPK pathway in A549 lung adenocarcinoma cells as well [74]. Zhao et al. [75] showed that curcumin treatment induced autophagy and suppressed invasion and proliferation in human melanoma cells via inhibition of the AKT/mTOR pathway.

## Ginsenoside

Kim et al. reported that ginsenoside Rg3 treatment of hepatocellular carcinoma, using a GFP-mRFP-LC3 construct, suppressed autophagic flux. Combined treatment with doxorubicin and ginsenoside Rg3 sensitized cells to death via inhibition of autophagic flux [76]. Ginsenoside 20(S)-Rg3 treatment of ovarian cancer cells inhibited cell growth and invasion while upregulating autophagic related molecules, such as LC3II, ATG5, and ATG7. Ginsenoside 20(S)-Rg3 treatment suppressed tumor growth while upregulating LC3II, ATG5, and ATG7 levels [46]. Zheng et al. [48] showed that ginsenoside RO (RO) treatment combined with chloroquine diphosphate did not enhance LC3 II accumulation in esophageal cancer, implying that RO inhibits autophagosome–lysosome fusion.

## Fisetin

Fisetin (3,3′,4′,7-tetrahydroxyflavone) is a flavonoid present in fruits and vegetables [77] that possesses anti-cancer [78], anti-inflammatory [79], and antioxidative [80] actions. Suh et al. [43] showed that fisetin treatment inhibited the growth of PC3Cap cells and induced LC3II autophagic marker expression via inhibition of mTOR1/2 signaling. Klimaszewska-Wisniewska et al. observed that combined treatment of fisetin and paclitaxel induced autophagic cell death in A549 non-small-cell lung cancer cells [81].

## Oridonin

Treatment with oridonin, an active diterpenoid compound isolated from *Rabdosia rubescens*, simultaneously induced apoptosis and autophagy via ROS in HeLa cells [82] and human multiple myeloma RPMI8266 cells [83]. Oridonin induced apoptosis and autophagy in human prostate cancer cells [84]. Treatment with the nitric oxide (NO) scavenger DTT and interruption of ERK and p53 activation by PD98059, pifithrin-α, or ERK siRNA decreased oridonin-induced apoptosis and autophagy. This indicates that NO/ERK/p53 inhibited oridonin-induced apoptosis and autophagy in L929 cells [85].

## Piperlonguminine

Piperlonguminine, from the long pepper (*Piper longum*), possesses anti-tumor activity [86] and suppresses the phosphorylation of AKT/mTOR, thereby upregulating ROS levels in several cancer cells, such as 786-O, PC-3, and MCF-7 cell lines [87]. Piperlongumine-induced autophagy depends on ROS activation [87]. Xiong et al. [88] also reported that piperlonguminine significantly induced apoptotic molecules such as Bax, Bcl-2, and caspase-3, autophagic markers such as Beclin-1 and LC3B, phosphorylation of p38 and JNK, and ROS levels in bone marrow mononuclear cells from patients with myeloid leukemia. Piperlonguminine activated p38 protein kinase via ROS, and SB203580, a p38 inhibitor, inhibited piperlonguminine-mediated autophagy [89].

## Honokiol

Honokiol, 2-(4-hydroxy-3-prop-2-enyl-phenyl)-4-prop-2-enyl-phenol), a small molecule polyphenol, induced both autophagy and apoptosis via inhibition of the PI3K/AKT/mTOR pathway in neuroblastoma cells [45]. Lu et al. showed that honokiol treatment of human thyroid cancer cells induced anti-tumorigenic effects in vitro and in vivo. LC3-II activity, a marker of autophagy, was increased by honokiol treatment in concentration- and time-dependent manners in thyroid cancer cells [90].

## Others

### Quercetin

Quercetin, a dietary antioxidant present in fruits and vegetables, is a chemopreventive compound. Wang et al. [42] reported that quercetin induced the appearance of autophagic vacuoles and formation of acidic vesicular organelles, with conversion of LC3-I to LC3-II via modulation of AKT/mTOR and hypoxia-induced factor 1α signaling in gastric cancer cells.

### [6]-Gingerol

Treatment with gingerol, which has anti-cancer effects, blocked autophagic flux, and gingerol combined with TRAIL sensitized cell death by blocking autophagic flux in A549 lung adenocarcinoma cells [91].

### Glabridin

Glabridin, an isoflavone, induced autophagy and apoptosis in Huh7 human liver cancer cells via activation of JNK1/2. Autophagy inhibitors, such as wortmannin and BafA1, enhanced glabridin-mediated apoptosis [92].

## Conclusions

The induction of autophagy is regarded as a strategy for cancer prevention because of its function as a tumor suppressor. Inhibition of the PI3K/AKT/mTOR signaling pathway, which is dysregulated in human tumors, affects autophagy. As shown in Fig. 2 and Table 1, natural agents regulate various autophagic signals. Therefore, finding natural agents that suppress PI3K/AKT/mTOR signaling or induce autophagy may be a good strategy for cancer chemoprevention.

**Table 1:** Mechanism of natural agents that induce autophagy and apoptosis in cancer cells

Natural agents	Cancer cell line	Pathway	References
Resveratrol	Ovarian cancer cells	STAT3	[52]
Ursolic acid	Prostate cancer cell	Beclin-1/Akt/mTOR	[62]
	Colorectal cancer cells with p53 mutant	JNK	[47]
	MCF-7 human breast cancer cells	EIF2AK3	[63]
	Cervical cancer cells TC-1	ATG5	[64]
Tanshione IIA	KBM-5 leukemia cells.	mTOR/p70S6K/AMPK	[68]
	Lung cancer 95D cells	ROS	[40]
Curcumin	Human colon cancer HCT116 cells	Transcription factor EB (TFEB)	[72]
	Human melanoma cells	AKT/mTOR	[75]
Ginsenoside 20(S)-Rg3	Ovarian cancer	LC3II, ATG5, and ATG7	[46]
Fisetin	Prostate cancer cells	mTOR	[43]
Oridonin	Human cervical carcinoma Hela cells	ROS	[82]
Honokiol	Neuroblastoma cells	PI3K/Akt/mTOR	[45]
Piperlongumine	Myeloid leukemias	P38/ROS	[88]

## Abbreviations

AMPK: AMP-activated protein kinase
CQ: chloroquine diphosphate
ER: endoplasmic reticulum
LC3: microtubule-associated protein light chain 3
mTOR: mechanistic target of rapamycin
NSCLC: non-small-cell lung cancer
PI3K: class III phosphatidylinositol 3-kinase
ROS: reactive oxygen species
STIM1: stromal interaction molecule 1
